# Early dysregulation of cardiac-specific microRNA-208a is linked to maladaptive cardiac remodelling in diabetic myocardium

**DOI:** 10.1186/s12933-019-0814-4

**Published:** 2019-01-29

**Authors:** Shruti Rawal, Prashanth Thevakar Nagesh, Sean Coffey, Isabelle Van Hout, Ivor F. Galvin, Richard W. Bunton, Philip Davis, Michael J. A. Williams, Rajesh Katare

**Affiliations:** 1 Department of Physiology-HeartOtago, Otago School of Medical Sciences, University of Otago, 270, Great King Street, Dunedin, 9010 New Zealand; 2 Department of Microbiology & Immunology, Otago School of Medical Sciences, University of Otago, Dunedin, New Zealand; 3 Department of Medicine, Dunedin School of Medicine, University of Otago, Dunedin, New Zealand; 4 Department of Cardiothoracic Surgery, Dunedin School of Medicine, University of Otago, Dunedin, New Zealand; 5 Present Address: New York University, New York, USA

**Keywords:** Diabetic heart disease, microRNA, Cardiac hypertrophy, Modulation of microRNA

## Abstract

**Background:**

The diabetic heart undergoes remodelling contributing to an increased incidence of heart failure in individuals with diabetes at a later stage. The molecular regulators that drive this process in the diabetic heart are still unknown.

**Methods:**

Real-time (RT) PCR analysis was performed to determine the expression of cardiac specific microRNA-208a in right atrial appendage (RAA) and left ventricular (LV) biopsy tissues collected from diabetic and non-diabetic patients undergoing coronary artery bypass graft surgery. To determine the time-dependent changes, cardiac tissue were collected from type 2 diabetic mice at different age groups. A western blotting analysis was conducted to determine the expression of contractile proteins α- and β-myosin heavy chain (MHC) and thyroid hormone receptor-α (TR-α), the negative regulator of β-MHC. To determine the beneficial effects of therapeutic modulation of miR-208a, high glucose treated adult mouse HL-1 cardiomyocytes were transfected with anti-miR-208a.

**Results:**

RT-PCR analysis showed marked upregulation of miR-208a from early stages of diabetes in type 2 diabetic mouse heart, which was associated with a marked increase in the expression of pro-hypertrophic β-MHC and downregulation of TR-α. Interestingly, upregulation of miR-208a preceded the switch of α-/β-MHC isoforms and the development of diastolic and systolic dysfunction. We also observed significant upregulation of miR-208a and modulation of miR-208a associated proteins in the type 2 human diabetic heart. Therapeutic inhibition of miR-208a activity in high glucose treated HL-1 cardiomyocytes prevented the activation of β-MHC and hence the hypertrophic response.

**Conclusion:**

Our results provide the first evidence that early modulation of miR-208a in the diabetic heart induces alterations in the downstream signaling pathway leading to cardiac remodelling and that therapeutic inhibition of miR-208a may be beneficial in preventing diabetes-induced adverse remodelling of the heart.

## Background

Cardiac remodelling is defined by the process of left ventricular (LV) adaptation to different forms of physiological and pathological stress [1]. At the molecular level, pathological stress induces cardiac gene reprogramming, i.e., re-expression of a battery of fetal cardiac genes while downregulating adult cardiac genes which subsequently leads to cardiac remodelling and heart failure [2, 3]. Histopathologically, cardiac remodelling in response to injury or stress typically involves a change in myocyte size (hypertrophy), reduction in proliferation of cardiomyocytes while activation in the proliferation of fibroblasts along with uncontrolled deposition of extracellular matrix (ECM) proteins results in dramatic cell death (apoptosis) [4]. Cardiomyocyte hypertrophy, fibrosis, apoptosis, and arrhythmias are the typical hallmarks of cardiac remodelling in the diabetic heart, and recent studies have shown that a series of molecular and structural alterations in the diabetic myocardium leads to a global deterioration of cardiac function [1–3].

Cardiac hypertrophy is an important compensatory mechanism of the heart in response to diverse pathophysiological stimuli and imbalance in hormonal signalling as observed in diabetic stress. In response to hyperglycaemic stress, the heart undergoes extensive cardiac remodelling, resulting in pathological growth of cardiomyocytes (hypertrophy) [1, 5]. Initially, this response aims to normalize wall stress and preserve contractile performance, but chronically it produces hypertrophy and may eventually lead to heart failure.

Although various pathways provide coordinated control of the hypertrophic process, little is known about their underlying molecular mechanisms. Exposure of the heart to stressors such as hyperglycaemia, haemodynamic overload, mechanical overload or ischemia may lead to cardiac remodeling with a change in the gene expression profile and a detrimental outcome. MicroRNAs (miRNAs) are established as the critical molecular modulators of cardiovascular development/function and pathological processes including cardiac hypertrophy [6, 7]. Indeed, downregulation of miR-133a has been previously reported to augment cardiomyocyte hypertrophy through serum- and glucocorticoid-inducible kinase 1 and Insulin-like growth factor 1 receptor mediated pathways [8]. Contrary to this, Van Rooij et al. did not find any of the morphological changes in cardiomyocytes associated with hypertrophic growth following miR-133a overexpression [9]. Therefore, it is essential to determine  the miRNA which has a direct role in regulating myocardial hypertrophy and those which exhibit altered expression secondary to hypertrophic growth in the diabetic myocardium.

Here, we demonstrate dysregulation of miR-208a in the human diabetic myocardium. miR-208a is encoded by an intron of the α-myosin heavy chain (MHC) gene which also encodes a major cardiac contractile protein [9]. Moreover, using a mouse model of type 2 diabetes, we confirmed that dysregulation of miR-208a begins from the early stages of the disease and that it precedes the development of hypertrophy in the diabetic heart. Finally, we also demonstrate that in vitro normalization of miR-208a in high glucose treated adult mouse cardiomyocytes prevented the activation of hypertrophic signals in these cells.

## Methods

### Ethics

The human myocardium study was approved by the Health and Disability Ethics Committee of New Zealand and the Human Ethics Committee at the University of Otago, New Zealand. All the patients provided written consent for the collection and use of samples in this study. The animal study using type 2 diabetes was approved by the Animal Ethics Committee at the University of Otago, New Zealand.

### Human myocardial tissue collection

Right atrial appendage (RAA) and epicardial left ventricular (LV) biopsies were collected from type-2 diabetic ischaemic heart disease (D-IHD) (n = 8–10) and non-diabetic ischaemic heart disease (ND-IHD) (n = 10) patients with preserved ejection fraction undergoing on-pump coronary artery bypass graft surgery. Myocardial tissue samples from non-diabetic healthy (ND-H, n = 5) were collected from cadavers without any known history of ischemic heart disease to be used as healthy controls. Tissue samples were snap-frozen and stored at − 80 °C immediately after collection for molecular analysis.

### Animal model of type 2 diabetes

BKS.Cg-m +/+Leprdb/J mice (db/db) and their non-diabetic C57BL/ksJ-lepr + littermates (db/+) (Jackson laboratory), were used as the model of type 2 diabetes and age-matched controls respectively. Under terminal anaesthesia, myocardial tissue samples were collected from both the groups at 7 different time points (8, 12, 16, 20, 24, 28, 32 weeks (W) of age, n = 10 each. Immediately after excision, the hearts were snap frozen in liquid nitrogen for molecular analyses.

### Echocardiography

Evolution of cardiac dysfunction in the diabetic myocardium was monitored every 4 weeks from 8 to 32 weeks of age in mice by measuring the changes in cardiac function using a Vivid E9 cardiovascular ultrasound system (GE Vingmed Ultrasound, Horten, Norway). Left ventricular wall thickness, systolic function (internal diameters, ejection fraction, fractional shortening) and diastolic function (E/A ratio, deceleration time (DecT)) were assessed as previously described [10–13]. LV mass was calculated from both mouse and human echocardiography data using the following equation: LV mass (mg) = [0.8 (1.04[([LVAWd or IVSd + LVIDd + LVPWd]^3^ − LVIDd^3^)]) + 0.6], where, LVAWd is left ventricular (LV) anterior wall thickness during diastole, IVSd is interventriculat septal thickness during diastole, LVIDd is LV internal diameter during diastole and LVPWd is LV posterior wall thickness during diastole [14].

### Molecular analyses

#### Total RNA isolation and quantitative real-time RT-PCR analysis

Total RNA was isolated from snap-frozen myocardial tissues and HL-1 cardiomyocytes (vide infra) using QIAGEN miRNeasy^®^ Mini kit as per manufacturer’s instructions [15–17]. Twenty nanograms of tissue/cell-derived total RNA was reverse transcribed using miR-208a and U6 (internal control) specific stem-loop structure and reverse transcription primers (Thermofisher, NZ). Following reverse transcription, specific Taqman hybridization probes were used to quantify the expression of miR-208a (Thermofisher, NZ). miR-208a expression was normalized to the internal control U6.

#### Western blot analysis

Western blotting for human and mice myocardial tissue homogenates and HL-1 cardiomyocytes was performed to study the expression of α- and β-MHC, the target proteins for miR-208a. In brief, the tissue/cells were homogenized in ice-cold RIPA buffer, proteins resolved by SDS-PAGE and transferred onto PVDF membrane. Membranes were probed with mouse anti-α-MHC (1:1000 dilution), mouse anti-β-MHC (1:1000 dilution), goat anti-thyroid hormone receptor- α (1:500 dilution, all from Abcam) and mouse anti-β-actin (1:1000 dilution, Santa Cruz Biotechnology) [6, 16, 18]. Due to the limitation in the size of the human myocardial tissue samples, western blot analysis in the human tissue samples was limited to RAA only. For the mice tissue, western blot analysis was performed on samples collected at 8, 16 and 32 weeks of age.

#### In vitro cell culture and high glucose experiments

HL-1 adult cardiomyocytes were a kind gift from Prof William Claycomb of Louisiana State University and were cultured in Claycomb medium according to the developer’s instructions [19]. For the experiments, cells (3 × 10^5^/well in 6-well plate or 1 × 10^4^/well in 96 well plate or 1 × 10^4^/well in 8-chamber slide) were exposed to high d-glucose (HG, 30 mM) or d-mannitol (NG, 30 mM, used as the osmotic control) for 24 h and 48 h. For longer high glucose experiments, the media was replaced with fresh supplemented Claycomb medium containing d-glucose (30 mM) or d-mannitol (30 mM) every 24 h [11, 20].

#### miR mimic and inhibitor (anti-miR) transfection

Following 24 h or 48 h high glucose exposure, HL-1 cardiomyocytes were transfected with miR-208a mimics/inhibitors/scrambled sequence using lipofectamine RNAiMAX (Life Technologies) according to the manufacturer’s instructions [13, 17, 18]. Briefly, for overexpression, cells cultured in normal glucose were transfected for 24 h with miR-208a mimic (5 pmol). Scrambled sequence (5 pmol) was used as negative controls. To silence the activity of miR-208a, cells cultured in both normal and high glucose were transfected with anti-miR-208a (5 pmol) or a scrambled sequence (5 pmol). For both the experiments, cells were incubated at 37 °C for further 24 h post transfection and samples were isolated at the end of the treatment period for molecular and histological analysis.

#### Immunocytochemistry

For immunocytochemistry, HL-1 cardiomyocytes were grown on 8-chamber slides and exposed to high glucose (30 mM) for 24 h, followed by transfection with miR-208a inhibitor/mimic/scrambled control. At the end of the treatment period, cells were fixed with freshly prepared 4% paraformaldehyde. Following serial washing and blocking with 10% goat serum, cells were incubated with primary antibody against α-actinin overnight at 4 °C  to stain the cardiomyocytes, followed by secondary antibody for another 30 min at RT. Finally, cells were counter-stained with DAPI (1:1000 for 3 min) to stain the nuclei. After the final wash with PBS, coverslips were mounted on glass chamber slides using Fluromount-G (Southern Biotech™) and allowed to dry for 24 h [21, 22]. α-actinin stained cardiomyocytes were captured in 3 random fields per chamber using a fluorescence microscope (Olympus BX51) at 20× magnification. After setting the threshold, areas of at least 15 cells per field were outlined and bucket-filled in Gimp. Cardiomyocyte area was calculated using the ImageJ Particle Analyzer algorithm [21]. Adobe Photoshop was utilized to compose and overlay the images (Adobe CS6).

### Statistical analysis

Data are presented as mean ± SEM. Comparisons between the groups were made using analysis of variance (ANOVA) followed by Tukey’s test for multiple comparisons. Unpaired *t* test was used to compare two groups.

Correlations between miR-208a and LV mass was done using Pearson′s correlation equations. A probability value (P value) less than 0.05 was considered statistically significant.

## Results

### Diabetes dysregulates the expression of miR-208a in the human heart

**Fig. 1:**
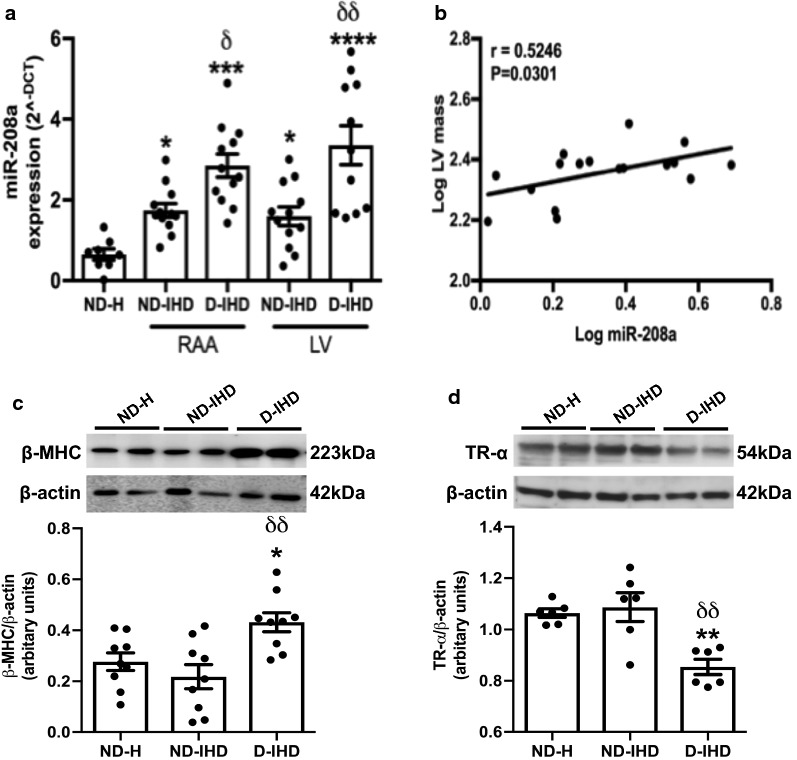
Dysregulated miR-208a expression in the diabetic heart. **a** Quantitative scatter plot with a bar graph showing the expression of miR-208a by RT-PCR analysis in the myocardial tissues collected from patients undergoing coronary artery bypass graft surgery or the cadavers. **b** Scatter plots showing the Pearson correlation coefficient of LV mass vs. miR-208a in all the study participants irrespective of the age or diabetes. There was a significant positive correlation (r = 0.5262, P = 0.03) between LV mass and miR-208a. **c**, **d** Quantitative scatter plot with a bar graph showing the expression of β-MHC (**c**) and TR-α (**d**) by western blot analysis in the myocardial tissues collected from patients undergoing coronary artery bypass graft surgery or the cadavers. Data for **a**–**d** are represented as mean ± SEM and n = at least 8 samples in each group for RT-PCR analysis and 5 for western blot. *P < 0.05 and ***P < 0.001 vs. non-diabetic healthy group (ND-H); ^δ^P < 0.05 and ^δδ^P < 0.01 vs. non-diabetic ischemic heart disease group (ND-IHD). One-way ANOVA was used for comparison between groups followed by Tukey’s test for multiple comparisons

Clinical characteristics of the study participants is illustrated in Table 1. Diabetic patients had significantly higher HbA1c compared to the non-diabetic patients undergoing surgery. Quantitative RT-PCR analysis showed significant upregulation of miR-208a in both RAA and LV regions of human type 2 diabetic myocardium in patients with IHD (P < 0.0001 vs. ND-IHD, Fig. 1a). Intriguingly, while ischemia alone (ND-IHD) significantly upregulated the expression of miR-208a (P < 0.001 and P < 0.0001 vs. ND-H, Fig. 1a), diabetes further upregulated the expression level of miR-208a in both RAA and LV (P < 0.05 and P < 0.01 vs. ND-IHD, Fig. 1a), thereby demonstrating the independent detrimental effects of diabetes on myocardium. Our previous study showed a similar increase in miR-208a in human type-2 diabetic heart with normal ejection fraction (EF) [23], however,  this study also included samples from patients with reduced EF and myocardial samples from those died due to non-cardiac reason. Echocardiography showed a non-significant increase in LV mass in the diabetic heart (P = 0.07, Table 1), however, pearson correlation analysis showed a singificant positive correlation between miR-208a and LV mass irrespective of diabetes (P = 0.03, Fig. 1b). Diabetic heart also showed a marked upregulation of pro-hypertrophic protein β-MHC (Fig. 1c) while its negative regulator TR-α was downregulated (Fig. 1d).

**Table 1:** Clinical characteristics and cardiac function measured by echocardiography in study participants

Sample ID	Age (years)	Sex	Diabetes duration (years)	HbA1c (mmol/mol)	BMI (Kg/m^2^)	Systolic blood pressure (mmHg)	Diastoic blood pressure (mmHg)	EF (%)	E/e’	E/A	IVSd (cm)	LVIDd (cm)	LVIDs (cm)	LVPWd (cm)	LV mass (gm)
Non-diabetic-IHD															
148	54	M	–	27	24.78	115	70	54	5.38	1.06	1.3	5.0	–	1.3	262
132	79	M	–	39	27.18	118	68	33	1.75	14	1.2	5.3	4.4	0.8	200
154	81	M	–	40	23.27	140	70	50	–	0.63	1.0	5.4	3.2	1.2	235
286	80	M	–	35	26.61	107	66	62.6	14.15	0.91	1.1	4.6	3.1	1.0	170
235	62	M	–	30	28.11	148	90	57	–	0.49	1.3	5.0	3.5	1.2	248
134	68	M	–	N/A	32.15	115	60	56	21.32	0.7	1.4	3.6	2.4	1.2	160
238	79	F	–	37	21.72	156	86	73	12.73	0.61	1.3	3.7	2.1	1.2	157
237	61	M	–	33	30.86	160	72	44	9.71	1.15	1.6	4.6	3.7	1.1	243
173	57	M	–	N/A	32.19	115	62	45	9.72	0.65	1.0	6.1	4.3	0.8	222
236	78	M	–	N/A	28.20	126	78	52.4	N/A	N/A	1.3	4.6	3.5	1.4	243
Average	69.90			34.43	27.51	130.00	72.20	52.70	10.68	2.24	1.25	4.79	3.36	1.12	214
SD	10.65			4.76	3.58	19.33	9.77	10.91	6.31	4.41	0.18	0.76	0.77	0.20	39.4
Diabetic-IHD															
221	54	M	6	84	26.73	130	72	55	7.85	1.06	1.1	5.1	3.3	1.3	N/A
293	54	M	3	47	34.66	115	65	66.4	8.77	0.71	1.2	5.0	3	1.2	234
174	79	M	58	73	20.98	120	50	28	15.59	0.51	1.4	5.3	4.2	1.2	287
177	81	M	10	39	21.07	130	95	27		0.76	1.2	5.0	4.1	1.3	248
229	85	M	28	42	24.16	105	60	67	22.02	0.77			2.8		
190	80	M	8	48	30.61	135	75	52	9.6	0.61	1.6	4.1	3.2	1.4	241
92	62	M	4	53	36.50	138	78	54	–	–	1.7	3.8	3.1	1.3	217
120	68	M	2	51	26.85	120	80	53	7.71	0.62	1.7	4.6	2.1	1.6	330
164	61	M	30	50	33.02	130	75	52	10	0.73					
301	78	M	11	36	32.81	128	78	72	–	0.66	1.8	4.0	2.3	1.3	245
Average	70.20		16.00	52.3**	28.74	125.10	72.80	52.64	11.65	0.71	1.51*	4.54	3.00	1.33*	257
SD	11.79		17.76	15.04	5.59	10.02	12.26	15.07	5.30	0.15	0.27	0.57	0.71	0.13	38.4
Non-diabetic vs. diabetic	0.9531			0.009	0.565	0.486	0.905	0.992	0.761	0.314	0.023	0.0231	0.4799	0.322	0.1204

*IHD* ischaemic heart disease, *BMI* body mass index, *EF* ejection fraction, *IVSD* intraventricular septal thickness at the end of diastole, *LVIDd* left ventricular (LV) internal diameter at the end of diastole, *LVIDs* LV internal diameter at the end of systole, *LVPWd* LV posterior wall thickness at the end of diastole, *N/A* data not available

*P < 0.5 vs. non-diabetic IHD

### Dysregulation of miR-208a in the diabetic myocardium is linked to progressive cardiac dysfunction

**Fig. 2:**
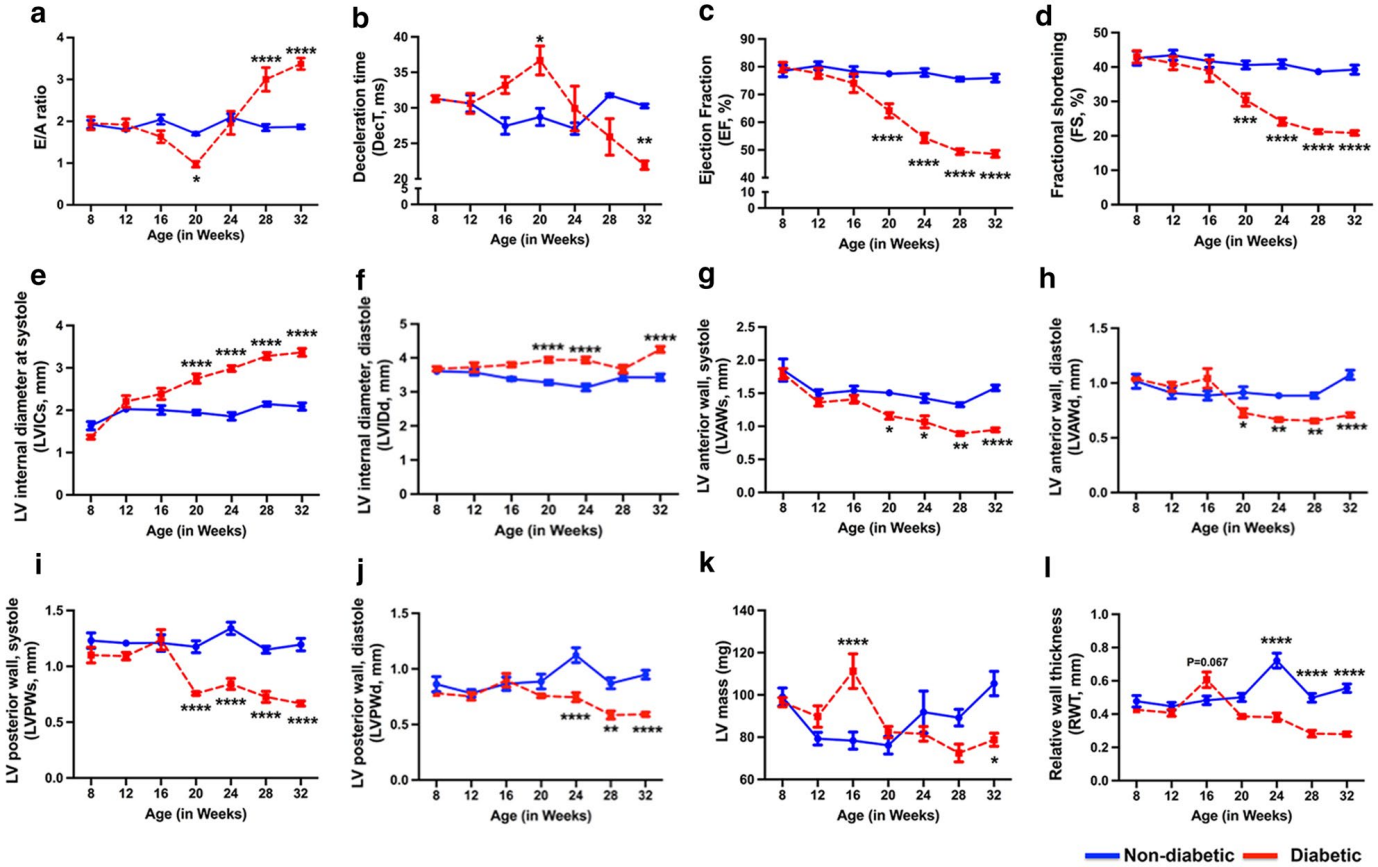
Progressive cardiac dysfunction in type 2 diabetic heart. Quantitative line graphs showing measures of diastolic function [E/A ratio (**a**) and deceleration time (DecT, **b**)], systolic function (ejection fraction (**c**), fractional shortening (**d**) and left ventricular (LV) internal diameter at systole (**e**) and diastole (**f**)) and wall thickness, (LV anterior wall at systole (**g**) and diastole (**h**), LV posterior wall at systole (**i**) and diastole (**j**), LV mass (**k**) relative wall thickness (**l**). Data are represented as mean ± SEM and n = 8 samples in each group. Two-way ANOVA was used for comparisons between groups at different time points, followed by Tukey’s test for multiple comparisons. *P < 0.05, **P < 0.01, ***P < 0.001 and ****P < 0.001 vs. non-diabetic heart of the corresponding age group

Having demonstrated the dysregulation of cardio-enriched miR-208a in the human diabetic myocardium, our next question was to determine if there is any correlation of miR-208a and cardiac dysfunction. For this, we used a mouse model of type 2 diabetes and serially monitored the changes in cardiac function, miR-208a expression and its target proteins α- and β-MHC and TR-α expression. Echocardiography analysis showed a significant decrease in E/A ratio at 20 W (P < 0.0001, Fig. 2a). Furthermore, a pseudonormalized pattern of diastolic dysfunction was observed at 24 W, evident by E/A ratio > 1 along with decrease in DecT time when compared to age-matched non-diabetic littermates (Fig. 2a, b). Importantly, a restrictive filling pattern of LV was observed in 28 W and 32 W old diabetic mice, evident by a significant increase in E/A ratio > 2 (P < 0.001 vs. non-diabetic, Fig. 2a) as well as a marked reduction in DecT (P < 0.001 vs. non-diabetic, Fig. 2b).

M-mode echocardiography analysis revealed the onset and progression of LV systolic dysfunction in type 2 diabetic mice as evidenced by reduced ejection fraction (EF, Fig. 2c), fractional shortening (FS, Fig. 2d) and increased LV internal diameter at systole (LVIDs, Fig. 2e) and diastole (LVIDd, Fig. 2f) from 20 W of age worsening with the evolution of the disease (P < 0.001 vs. age-matched non-diabetic mice, Fig. 2c–f). There was a significant increase in LV mass in db/db mice at 16 W (Fig. 2k). With the progression of the disease there was a gradual decrease in anterior wall thickness during systole (LVAWs, Fig. 2g) and diastole [LVAWd, Fig. 2h)], posterior wall thickness in systole (LVPWs, Fig. 2I) and diastole [LVPWd, Fig. 2j)] and LV mass (Fig. 2k). This was also accompanied by a decrease in relative wall thickness (RWT) in diabetic mice, an index of cardiac remodeling (Fig. 2l).

**Fig. 3:**
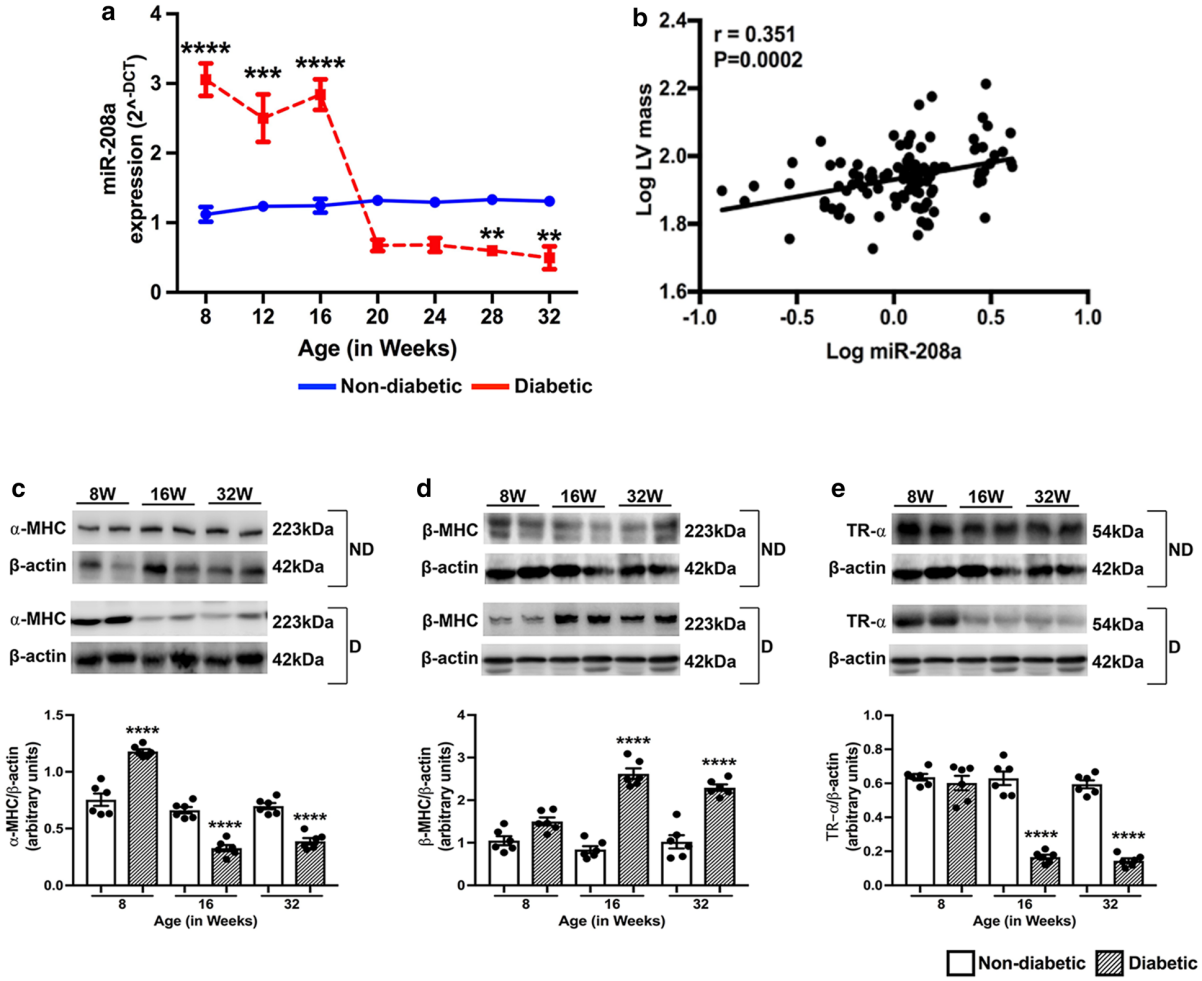
LV hypertrophy and remodelling of the diabetic heart. **a** Quantitative line graphs showing the expression of miR-208a by RT-PCR analysis in the myocardium of type 2 diabetic and the age-matched non-diabetic mice at different time points. **P < 0.01, ***P < 0.001 and ****P<0.0001 vs. non-diabetic heart of the corresponding age group. **b** Scatter plots showing the Pearson correlation coefficient of LV mass vs. miR-208a in all the study animals irrespective of the age or diabetes. There was a significant positive correlation (r = 0.351, P = 0.0002) between LV mass and miR-208a. **c**–**e** Representative immunoblots and quantitative bar graphs showing the expression level of α-MHC (**c**), β-MHC (**d**) and TR-α (**e**) protein expression in type-2 diabetic mouse heart collected at 8, 16 and 32 weeks of age. Data are Mean ± SEM and n = 6 samples in each group. Two-way ANOVA was used for comparisons between groups at different time points in mice heart, followed by Tukey’s test for multiple comparisons. ****P < 0.0001 vs. age-matched non-diabetic control

To determine if changes in cardiac function are associated with dysregulation in the expression pattern of miR-208a, RT-PCR analysis was conducted at each time point. As shown in Fig. 3a, there was a marked upregulation of miR-208a in the diabetic heart as early as 8-weeks of age (Fig. 3a), when echocardiography showed normal cardiac function (Fig. 2). Intriguingly, miR-208a remained upregulated until the 16 W time point (Fig. 3a) after which there was a significant downregulation in its expression (Fig. 3a). The initial upregulation of miR-208a in the mouse  diabetic heart was consistent with its upregulation in expression which was  observed in human myocardium (Fig. 1a). Interestingly, like in the human heart, there was a significant positive correlation between miR-208a and LV mass, further supporting the role of miR-208a in hypertrophy (Fig. 3b).

### miR-208a is involved in LV hypertrophy and remodelling of the diabetic heart

To determine the functional significance of the differential expression of miR-208a, we next measured the expression levels of the contractile proteins α- & β-MHC in the type 2 diabetic mice hearts at 8 weeks, 16 weeks and 32 weeks of age by western blot analysis. β-MHC is a slow myosin contractile protein, and its increased expression is a common feature of pathological cardiac hypertrophy and remodelling.

Results showed a significant increase in α-MHC protein expression as early as 8 weeks in the diabetic heart (Fig. 3c) corresponding to an increase in expression of miR-208a (p < 0.0001 vs. non-diabetic, Fig. 3a). Follow-up analysis showed a significant decrease in the expression of α-MHC at 16 and 32 weeks of age in the diabetic heart (Fig. 3d). This was associated with a significant increase in the expression of pro-hypertrophic protein β-MHC at 16 and 32 weeks of age (Fig. 3e), indicating myosin switching in the contractile apparatus. Interestingly, we observed a sustained expression of miR-208a even after downregulation of α-MHC in the diabetic heart at 16 weeks of age  (Fig. 3a) which is in agreement with a previous report, suggesting a longer half-life of miR-208a relative to α-MHC [9]. Further, we also observed a marked decrease in the expression level of thyroid hormone receptor-α (TR-α) in the diabetic heart (Fig. 3d). Previous studies have suggested that miR-208a potentiates βMHC expression through a mechanism involving the TR-α. Of note, the marked upregulation of miR-208a in diabetic mice preceded the switch of α/β-MHC isoforms as observed at 16 weeks in the diabetic heart (Fig. 3).

### Inhibition of miR-208a ameliorates cardiac hypertrophy in high glucose treated HL-1 mouse cardiomyocytes

**Fig. 4:**
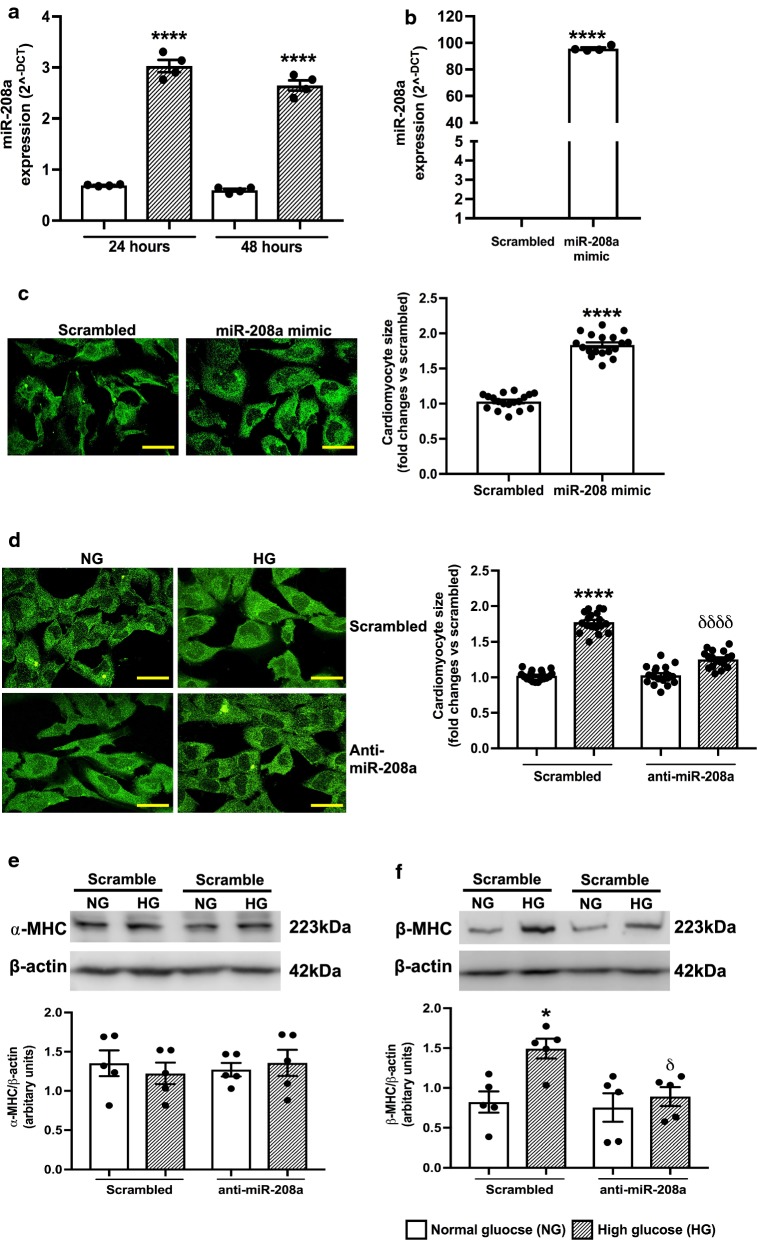
Inhibition of miR-208a ameliorates cardiac hypertrophy in high glucose treated HL-1 mouse cardiomyocytes. **a**,** b**: Quantitative scatter plot with a bar graph showing the expression of miR-208a by RT-PCR analysis in normal and high glucose treated HL-1 cardiomyocytes at 24 and 48 h (**a**) and 24 h after transfection with miR-208a mimic (**b**). Data are represented as mean ± SEM. ****P < 0.0001 vs. normal glucose (NG) treated cells (**a**) or scrambled treated cells (**b**). **c**,** d**: Representative fluorescence microscopic images and quantitative scatter plot with a bar graph showing the changes in cardiomyocytes size after treatment with miR-208a mimic (**c**) or anti-miR-208a (**d**). Data are represented as mean ± SEM. ****P < 0.0001 vs. scrambled treated group and δδδδP < 0.0001 vs. high glucose (HG)-scrambled sequence treated group. **e**, **f**: Representative immunoblots and quantitative bar graphs showing the expression level of α-MHC (**e**) and β-MHC (**f**) protein expression in HL-1 cardiomyocytes treated with normal or high glucose and transfected with either scrambled or anti-miR-208a. Data are Mean ± SEM. *P < 0.05 vs. NG-scrambled treated cells and ^δ^P < 0.05 vs. HG-scrambled treated cells. Two-way ANOVA was used for comparisons between groups with different treatments, followed by Tukey’s test for multiple comparisons (**a**, **d**, **e**, **f**) or unpaired student T-Test (**b**, **c**). ****P < 0.0001 vs. age-matched non-diabetic control

Finally, to determine if therapeutic modulation of miR-208a can ameliorate the detrimental effects of high glucose on cardiac hypertrophy, we conducted a gain or loss of function study using HL-1 adult cardiomyocytes. Treating HL-1 cardiomyocytes with high glucose for 24 h and 48 h showed a significant upregulation of miR-208a at both the time points (Fig. 4a). Therefore, the 24 h time point was chosen to carry out all subsequent experiments on HL-1 cells. This also confirms that response of HL-1 cardiomyocytes to high glucose is comparable to that  observed in type 2 diabetic mouse and human hearts.

To confirm the pro-hypertrophic effect of miR-208a in adult cardiomyocytes, we next overexpressed miR-208a in normal glucose cultured HL-1 cardiomyocytes using miR-208a mimic under normal conditions. One advantage of this strategy was that it enabled us to determine if cardiac hypertrophy is due to the direct effect of miR-208a overexpression or a result of HG stress. The RT-PCR analysis confirmed upregulation of miR-208a, 24 h after transfection (Fig. 4b). Importantly, overexpression of miR-208a was sufficient to provoke cardiomyocyte hypertrophy (Fig. 4c).

Next to determine if inhibition of miR-208a activity in the high glucose treated cardiomyocytes is beneficial, high glucose treated cardiomyocytes were transfected with anti-miR-208a to inhibit its activity. As expected, silencing miR-208a activity significantly attenuated the increase in surface area of HL-1 cardiomyocytes reflecting cardiomyocytes hypertrophy in vitro (Fig. 4d).

Further, western blot analysis revealed a significant increase in the expression of the β-MHC protein in high glucose treated HL-1 cardiomyocytes, while the expression of α-MHC remained unchanged (Fig. 4e). Interestingly, inhibition of miR-208a activity prevented this increase in β-MHC (Fig. 4f) which was in parallel to the reduction in cardiomyocyte hypertrophy (Fig. 4d). Together, these data suggest that upregulation of miR-208a was required to activate the switch in the isoforms arrangement from α- to β-MHC and therefore, contributed to the development of cardiomyocyte hypertrophy. This was consistent with the data shown in diabetic mice heart, where the upregulation of miR-208a at 16 weeks was sufficient to activate the switch of α to β-isoform of MHC at 20 weeks.

## Discussion

Results from our study has revealed the involvement of cardiac-enriched miR-208a in the progression of diabetes-mediated maladaptive cardiac remodelling and the causal relation between miR-208a and cardiac hypertrophy protein MHC using in vitro adult cardiomyocytes. These results are supported by similar changes in the human type 2 diabetic heart. Interestingly, ischemia alone significantly upregulated the expression of miR-208a which was consistent with other studies, showing an increased level of circulating miR-208a in human [24] and animals [25, 26] after myocardial infarction. Important finding in this study is that diabetes further increased the expression of miR-208a in the heart.

### Possible role for miR-208a in inducing upregulation of β-MHC

Cardiac contractility is typically maintained by a tight balance between the fast isoform of myosin heavy chain (α-MHC) and the slow isoform of myosin heavy chain (β-MHC) levels [27]. Of note, one of the molecular hallmarks of pathological cardiac hypertrophy and remodelling is the upregulation of β-MHC. Experimental studies have demonstrated the specific induction of β-MHC mRNA and protein levels by disease-related hypertrophic stimuli in vivo and by hypertrophic agonists in culture [28–30] but not by any physiological stimuli such as exercise [31]. A marked increase in the expression of β-MHC in the diabetic heart in our study, therefore, suggests the initiation of molecular alterations that induce hypertrophy. Previous reports demonstrated a crucial role for miR-208a in the stress-dependent hypertrophic growth of cardiomyocytes [9], and that miR-208a is essential to induce the upregulation of β-MHC by directly targeting thyroid hormone-associated protein 1 (THAP1) and myostatin, two negative regulators of muscle growth and hypertrophy [32]. However, this is the first study to demonstrate that marked upregulation of β-MHC is concordant with the upregulation of miR-208a in both the human and mouse type 2 diabetic heart. We further showed significant downregulation of TR-α, a downstream regulator of THAP1.

### Upregulation of miR-208a precede structural remodeling

Another interesting finding of the current study was that the upregulation of miR-208a in the type 2 diabetic mouse heart preceded the activation of the fetal cardiac gene program and was associated with a switch of α/β-MHC isoforms with ensuing marked cardiac dysfunction. These findings are in line with existing reports where it was shown that the switch of α/β-MHC isoforms occurs in diabetic myocardium before the echocardiographic detection of ventricular hypertrophy [33, 34]. Consistently, we observed a similar isoform switch in type 2 diabetic mouse heart, although there was no clear evidence for hypertrophy in the diabetic heart by echocardiography. A steep increase in LV mass at 16 weeks of age in the diabetic heart was suggesting the heart is undergoing hypertrophic remodelling, however, this need to be cautiously interpreted as we did not observe any significant changes in the wall thickness. In contrast, the functional data from the human participants were clearly pointing towards hypertrophic remodelling in the diabetic heart showing a significant increase in interventricular sepatal thickness and posterior wall thickness during diastole which was associated with marked increase in the LV mass. Moreover, both the mouse and human study showed a positive correlation between miR-208a and LV mass. While more studies are required, these data clearly suggest a critical role of miR-208a in remodeling of the diabetic myocardium. Furthermore, using the in vitro cardiomyocytes, we also confirmed that the regulation of β-MHC was directly linked to miR-208a expression level. The cellular content of β-MHC was specifically increased in hypertrophic cardiomyocytes, and therapeutic inhibition of miR-208a activity was able to attenuate not only the hypertrophic response but also decreased the expression of β-MHC. These observations provide new insight into the mechanistic role of miR-208a in promoting cardiac remodelling through β-MHC-dependent hypertrophic activity, indicating activation of fetal cardiac gene programming in diabetic cardiomyocytes.

### Mature miR-208a may have a longer half-life

Given that miR-208a is encoded by an intron of the α-MHC gene [35], its expression is dependent on the expression of α-MHC. Intriguingly, in our study, the expression of the α-MHC gene was decreased at 16 W in the type 2 diabetic mouse heart, while miR-208a remained upregulated. This observation suggests a longer half-life of mature miR-208a even after downregulation of its host gene. This was supported by an earlier time course study showing persistent expression of miR-208a even after the downregulation of α-MHC [9]. Furthermore, in the compensated phase of in vivo studies, the expression of miR-208a was downregulated in the diabetic heart, while the expression of β-MHC persistently remained at a higher level. While the precise reason for this discrepancy is not known, it is possible that the early upregulation of miR-208a in the diabetic heart leads to persistent downregulation of TR-α thereby releasing its inhibitory switch on β-MHC.

## Conclusion

In conclusion, we show that upregulation of α-MHC triggers the activation of miR-208a in the early stages of diabetes. Activated miR-208a induce alterations in the downstream signaling pathway, mediating the switch of α/β-MHC isoforms leading to the development of cardiac remodelling. These results, therefore, lay a foundation for the possible crucial role of miR-208a as an early molecular modulator of maladaptive remodelling in the diabetic myocardium. Future studies focusing on the beneficial effects of inhibition of miR-208a activity in vivo will aid in the translation of these findings to the clinic.

## Abbreviations

ANOVA: analysis of variance
D: diabetic
DecT: deceleration time
ECM: extra cellular matrix
EF: ejection fraction
HG: high glucose
IHD: ischaemic heart disease
LVAWd: left ventricular anterior wall thickness at end diastole
LVAWs: left ventricular anterior wall thickness at end systole
LVIDd: left ventricular internal diameter at end diastole
LVIDs: left ventricular internal diameter at end systole
LVPWd: left ventricular posterior wall thickness at end diastole
LVPWs: left ventricular posterior wall thickness at end systole
LV: left ventricular
MHC: myosin heavy chain
MiRNA: microRNA
mM: millimole
ND: non-diabetic
NG: normal glucose
pMol: picomole
RAA: right atrial appendage
RIPA buffer: radioimmunoprecipitation assay buffer
RT-PCR: real-time polymerase chain reaction
RWT: relative wall thickness
THAP1: thyroid hormone-associated protein 1
TR-α: thyroid hormone receptor-α
